# Tuberculosis care: an evaluability study

**DOI:** 10.1590/0104-1169.3294.2482

**Published:** 2014

**Authors:** Ardigleusa Alves Coelho, Cláudia Santos Martiniano, Ewerton Willian Gomes Brito, Oswaldo Gomes Corrêa Negrão, Ricardo Alexandre Arcêncio, Severina Alice da Costa Uchôa

**Affiliations:** 1 Doctoral student, Universidade Federal do Rio Grande do Norte, Natal, RN, Brazil. Professor, Departamento de Enfermagem, Universidade Estadual da Paraíba, Campina Grande, PB, Brazil; 2 Doctoral student, Universidade Federal do Rio Grande do Norte, Natal, RN, Brazil. Assistant Professor, Departamento de Saúde Coletiva, Universidade Federal do Rio Grande do Norte, Natal, RN, Brazil; 3 PhD, Professor, Escola de Enfermagem de Ribeirão Preto, Universidade de São Paulo, WHO Collaborating Centre for Nursing Research Development, Ribeirão Preto, SP, Brazil; 4 PhD, Associate Professor, Departamento de Saúde Coletiva, Universidade Federal do Rio Grande do Norte, Natal, RN, Brazil

**Keywords:** Public Health, Health Evaluation, Tuberculosis

## Abstract

**OBJECTIVE:**

to verify whether the tuberculosis control program (TCP) is evaluable and to examine the feasibility of building an evaluation model in apriority municipality for the control of tuberculosis.

**METHOD:**

this evaluability study was conducted in a municipality in northeastern Brazil. For data collection, documental analysis and interviews with key informants were performed. For indicator validation, the nominal group technique was adopted.

**RESULTS:**

the details of TCP were described, and both the logical model and the classification framework for indicators were developed and agreed up on, with the goal of characterizing the structural elements of the program, defining the structure and process indicators, and formulating the evaluation questions.

**CONCLUSION:**

TCP is evaluable. Based on logical operational analysis, it was possible to evaluate the adequacy of the program goals for the control of tuberculosis. Therefore, the performance of a summative evaluation is recommended, with a focus on the analysis of the effects of tuberculosis control interventions on decreasing morbidity and mortality.

## Introduction

In the last decade, the incidence and mortality rates of tuberculosis (TB) have decreased in the six geographical regions defined by the World Health Organization (WHO) and in most of the 22 countries that account for 80% of TB cases worldwide, among which Brazil occupies the 19^th^ position. TB represents a serious public health problem, with approximately 9 million new cases and 1.5 million deaths each year^(^ 1 ^)^. In Brazil, TB kills 4500 people annually and is the leading cause of death among HIV/AIDS patients^(^ 2 ^)^. Despite the reduction in the number of cases in Brazil, the incidence rates have increased substantially among vulnerable population groups living in large cities^(^ 3 ^)^.

TB has assumed the status of a global emergency^(^ 1 ^)^ and has been included in the government's policy agenda in several countries. To achieve the goal of decreasing the global burden of TB (incidence and mortality) by up to 50% by 2015 in comparison with the 1990 rate, the Ministry of Health of Brazil-through the National Tuberculosis Control Program (NTCP) and guided by the STOP-TB strategy-has endorsed the implementation and sustainability of the directly observed treatment short-course (DOTS) to improve laboratory diagnosis, conduct supervised treatments with the continuous supply of drugs, and develop an adequate information and registry system for program monitoring^(^ 2 ^,^ 4 ^-^ 6 ^)^.

In conjunction with the national primary health care policy, the NTCP aims to intensify the decentralization of activities associated with the diagnosis and treatment of TB in primary health care since 2006. Furthermore, it aims to strengthen social control mechanisms and ensure the sustainability of disease control actions^(^ 2 ^)^. Considering the epidemiological complexity of TB and the challenges imposed on the health care system and services for the eradication of the disease in the 21^st^ century, the evaluation of the tuberculosis control program (TCP) preceded by an evaluability study (ES) becomes relevant. In this context, ES is an important procedure to determine the feasibility of a systematic evaluation of TCP performance^(^ 7 ^)^. Internationally, the use of ES in different programs, disciplines, and contexts is evident^(^ 8 ^)^. A previous study conducted in Canada^(^ 9 ^)^ examined the feasibility of evaluating a program for survivors of torture. With regard to tuberculosis, the use of a logical model of the community action program for the prevention of TB in public health in Ontario, Canada, was mentioned when reporting the use of an intervention based on the Ottawa Charter for Health Promotion^(^ 10 ^)^.

In Brazil, studies on public health are scarce. Relevant studies include topics on human resource policies^(^ 11 ^)^ and women's health^(^ 12 ^)^. Studies focused on tuberculosis include the evaluation of TB control interventions established in Niterói, Rio de Janeiro^(^ 13 ^)^, and in the legal Amazon^(^ 14 ^)^. Therefore, this study aimed to verify whether the TCP is evaluable, verify the feasibility of building an evaluation model of the program, and propose recommendations for future evaluation studies.

## Methods

This evaluability study or pre-evaluation^(^ 7 ^)^ examined the feasibility of building an evaluation model for TCP and was conducted between August 2011 and June 2012 in the reference health unit for TB control in Campina Grande, state of Paraíba (PB), Brazil. In accordance with Resolution 196/96 of the National Health Council, the study was approved by the Research Ethics Committee of the State University of Paraíba under Protocol number CAAE 0394.0.133.000.11.

Evaluability studies are essential to the description of specific programs by identifying goals, objectives, and actions; structuring the logical models based on resources, activities, expected impacts, and possible correlations between program components; formulating evaluative questions; designing evaluation models; identifying the entities involved or interested in the evaluations^(^ 9 ^)^; and proposing recommendations to the program and identifying the relevance of implementing such evaluations.

A logical model was developed to systematically identify the issues underlying the TCP by detailing the resources, activities, expected results, and correlations between these elements^(^ 7 ^)^. During the process of defining the logical model, it is important to review it and, if necessary, to readjust it to incorporate new aspects that were not previously addressed in its initial conception, with the goal of making it a valuable tool when defining the focus of the evaluation^(^ 15 ^)^.

During the building of the logical model, documental analysis and interviews with key informants were conducted. The documental analysis included institutional technical documents available on the website www.saude.gov.br the Recommendation Manual for TB Control^(^ 2 ^)^ and Directly Observed Treatment of Tuberculosis in Primary Health Care- Nursing Protocol^(^ 16 ^)^. These documents allowed understanding of the theory of the program by detailing the interventions, the management levels involved in program implementation, and the identification and analysis of the program components.

The evaluation of the draft version of the logical model aimed to identify its components and determine whether the proposed model adequately represented the program logic. The evaluation was performed in a reference health unit by a doctor and nurse specializing in TCP using semi-structured interviews, according to the guidelines proposed by McLaughlin and Jordan^(^ 17 ^)^.

After building the logical model, the classification framework for indicators was elaborated, and its criteria and indicators were identified using the nominal group technique^(^ 18 ^)^ with the help of eight TCP experts and researchers from the field of evaluation. Moreover, averages were calculated to attribute importance to each component analyzed, and the standard deviations (SD) were calculated to evaluate the degree of consensus for each criterion proposed.

The framework containing the evaluation questions was constructed based on the literature search and on the logical model. In the convergent phase, the questions raised were related to the aims of the evaluation (implementation phase of the TCP), considering both the questions initially formulated in the study and interviews with individuals interested in the evaluation (divergent phase of the TCP)^(^ 19 ^)^.

## Results

The diversity of organizational characteristics of access to tuberculosis diagnosis affect the proper management of the disease^(^ 20 ^)^ in local health services and strengthen the performance of evaluation studies centered in the operational aspects^(^ 2 ^)^ of the TCP. From this perspective, the city of Campina Grande-PB, where the evaluability study was conducted, is one of the priority cities for TB control in Brazil, and TB control interventions in Campina Grande are performed in family health units and by reference health unit. With regard to diagnosis and treatment, the centralization of diagnostic procedures by reference health units and the low effectiveness in the achievement of the directly observed treatment (DOT) by family health strategy (FHS) teams are evident.

The normative TCP documents analyzed to build the logical model and the recommendation manual for TB control provided details on the following program components: planning and administration, health care, strategic information, institutional and human development, and social communication and mobilization. In addition, the roles and responsibilities of each administration level of the Brazilian Unified Health System (*Sistema Ú*nico* de Saúde - *SUS)involved in TB control are described. During the construction of the logical model, the program's design, goals and actions aimed at controlling TB were discussed.

The program was designed as a set of "innovative strategies to expand and strengthen the DOTS, with a focus on coordinating this strategy with other government programs to further control TB and other comorbidities such as HIV/AIDS"^(^ 2 ^)^. Despite the decentralization of TB control in primary health care to ensure free access to diagnosis and treatment of TB patients, decentralization is not clearly stated among the components of the program.

**Figure 1:**
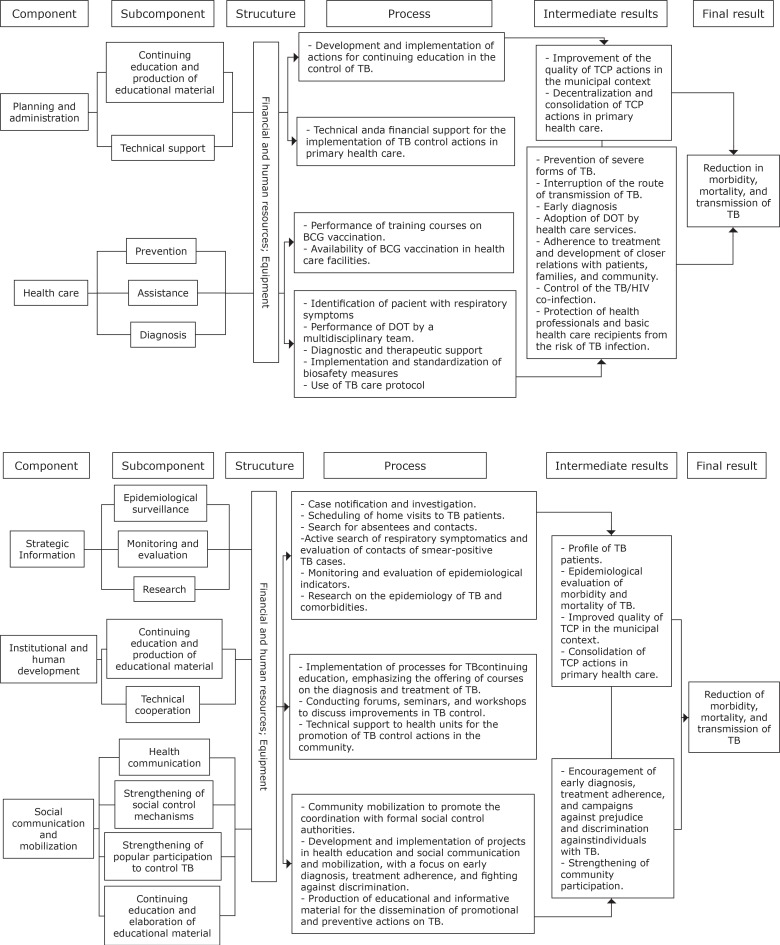
- Logical model of the tuberculosis control program in Campina Grande, Paraíba state, Brazil, in 2012

The logical model (Figure 1) allowed the identification of the activities to be implemented at the municipal level for each program component, to interrupt the route of TB transmission and to reduce morbidity and mortality. Certain specific elements were considered, including the duties and responsibilities of federal, state, and municipal authorities and the proposed structuring of health care for TB patients to correlate activities compatible with the five components with municipal management actions.

In addition to the subcomponents established by the program for the components 'health care' and 'strategic information', new subcomponents were added to the following components: Planning and Administration, Institutional and Human Development, and Social Communication and Mobilization (Figure 1) to characterize the structure available and the actions to be executed to achieve the aims and expected results. As the components of the logical model were presented, the flow of resources and results was determined using the following questions: do the resources planned allow the activity to be completed? Are the proposed activities tailored to the components? Do the proposed activities enable the achievement of the results? Considering the results achieved, does the program have an impact?

**Figure 2:**
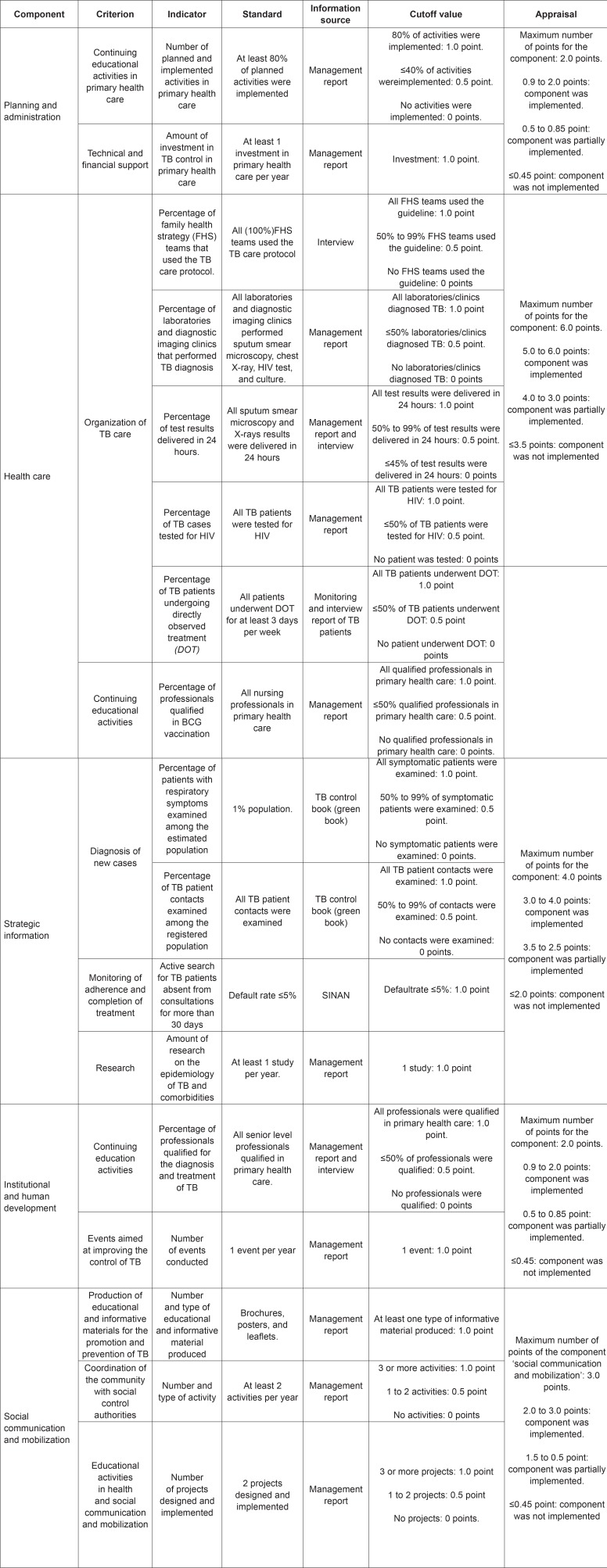
- Classification framework for indicators

The classification framework for indicators (Figure 2) was constructed considering that a program evaluation should be based on parameters^(^ 15 ^)^ and, for each model component, the criteria, indicators, and standards related to the structure and process were established. The evaluative questions derived from the analysis of the logical model and from the interviews were as follows: what can be changed in the local context to favor executing the intervention? Do the processes involved in the production and management of TB control actions as well as the results provide an adequate quality of health care?

## Discussion

This study highlights the need to incorporate new activities into the political and institutional context to meet internationally established goals^(^ 1 ^)^, with an emphasis on the effective decentralization of intervention strategies for family health care. The logical model can be used as a tool for the assessment and monitoring of the program and to evaluate its effects^(^ 21 ^-^ 22 ^)^. Accordingly, the elements addressed in each component indicate an interdependence that enables the achievement of the expected results. The proposal of subcomponents-continuing education and elaboration of instructional material, strengthening of social control mechanisms, and strengthening of popular participation to control tuberculosis-can improve the participation and effective coordination of the various authorities involved in TB control, including intersectoral actions for the creation of stronger bonds between patients and family health strategy teams^(^ 23 ^)^. The visual map of the TCP expands its scope and reinforces the use of the logical model proposed at the municipal level for the creation of an evaluation model in the context of primary health care that can improve control programs through including decision-making processes. Importantly, the proposed model can be reviewed to allow the incorporation of new aspects in the light of improvements in TB control policies.

Using the classification framework for indicators comprising criteria, indicators, and previously developed standards,​ it is possible to assess TCP to verify whether the intervention results are being achieved as planned and are reaching the target population^(^ 13 ^)^. Moreover, evaluation questions are decisive for the success of the evaluation by defining what topics will be evaluated in conjunction with the focus of the evaluation^(^ 15 ^)^.

## Conclusion

The tuberculosis control program is an evaluable program when considering its structural elements. Therefore, the definition of structure and process indicators can contribute to the production of knowledge, improve control measures, minimize the negative social image, and control damage. By comparing the logical model with reality, limitations are evident in the operationalization of health care with respect to the decentralization of basic health care. For this reason, it is important to objectively identify the means that are needed to achieve decentralization. For the remaining components, the operational logical analysis of the program indicates the adequacy of the goals proposed for the resolution of these issues. Furthermore, a summative evaluation focusing on the analysis of the effects of the intervention on the reduction of morbidity and mortality is recommended. We highlight the political and administrative difficulties in involving administrators in this study because their participation favors the critical analysis of the various stages of the study and affects the focus of the program.
